# Rethinking the Doses of Tissue Plasminogen Activator and Deoxyribonuclease Administrated Concurrently for Intrapleural Therapy for Complicated Pleural Effusion and Empyema

**DOI:** 10.7759/cureus.2214

**Published:** 2018-02-21

**Authors:** Ayoub Innabi, Alok Surana, Bashar Alzghoul, Nikhil Meena

**Affiliations:** 1 Internal Medicine, University of Arkansas for Medical Sciences, Little Rock, USA; 2 Pulmonary and Critical Care, University of Arkansas for Medical Sciences, Little Rock, USA

**Keywords:** empyema, fibrinolytics, surgery

## Abstract

Background

Complicated parapneumonic effusions empyema (CPEE) is fairly common and associated with increased morbidity and mortality. The Multicenter Intrapleural Sepsis Trial 2 (MIST 2) established the combination of intrapleural deoxyribonuclease (DNase) and tissue plasminogen activator (tPA) as an effective treatment for CPEE, thereby avoiding surgery and decreasing the length of hospitalization. MIST 2, however, used a labor-intensive protocol with some risk of bleeding. We hypothesize the simpler regimen of concurrent administration of intrapleural tPA and DNase (lower dose of tPA and a higher DNAse dose) to be equally effective with a decreased risk of bleeding.

Methods

Retrospective analysis of the concurrent administration of intrapleural tPA and DNase for CPEE during November 2014 to February 2016 was done at a tertiary care center. The inclusion criteria included 1) pleural fluid with any of the following: (a) exudative and loculated effusion in a patient with pneumonia, (b) gram stain/culture positive, (c) macroscopically purulent 2) chest tube placement during current hospitalization 3) concurrent administration of intrapleural tPA and DNase (4mg and 10mg per instillation respectively). The exclusion criteria was 1) chest tube placement prior to current hospitalization and 2) age < eighteen.

Results

Seventeen patients received concurrent tPA and DNase therapy for CPEE in the study period. Two had chest tubes placed prior to current hospitalization and were excluded. Twelve patients (80%) were successfully discharged with clinical resolution of CPEE with medical therapy. One (7%) patient required surgery. No mortality due to pleural sepsis was noted. The median number of concurrent tPA and DNase treatment was two. Median cumulative tPA dose was 8 mg (mean: 14.1±17 mg) and median cumulative DNase dose was 20mg (mean: 19.7 ± 12.2 mg). The median dwell time for the chest tubes was 8.5 days. Our regimen had similar success when compared to MIST 2 and allowed for lesser treatments and cumulative doses. No complication of intrapleural therapy with hemorrhagic conversion of CPEE, or worsening pain leading to discontinuation of therapy was noted.

Conclusion

The concurrent administration of intrapleural therapy at lower doses than the current standard MIST 2 protocol is practical, efficient and effective. We suggest a higher DNase dose with a lower tPA dose which may further decrease hemorrhagic complications. Further randomized trials are required to establish the optimal dose of intrapleural therapy for CPEE.

## Introduction

Complicated parapneumonic pleural effusion and empyema (CPEE) are common in hospitalized patients [1]. A significant number (20% to 40%) of hospitalized patients with pneumonia develop pleural sepsis [2], leading to higher mortality. Elderly, immuno-compromised patients and patients with multiple comorbidities contribute significantly to this mortality [1]. Prompt drainage is a cornerstone in the treatment of CPEE, but loculation and viscosity can make drainage difficult. Surgical drainage and decortications are required in the event of medical therapy failure, leading to greater morbidity and longer hospital stays [3-4]. The Multicenter Intrapleural Sepsis Trial 2 (MIST 2), demonstrated that combination of intrapleural therapy with tissue plasminogen activator (tPA) and deoxyribonuclease (DNase) improved drainage of infected effusions and reduced both the need for surgical intervention and the length of hospital stay [3].

The MIST 2 study consisted of 210 patients with pleural infection who were randomized to one of the four treatment arms: tPA and placebo, tPA and DNase, DNase and placebo, and double placebo. The dose of DNase given was 5 mg, and the dose of tPA was 10 mg. Medications were instilled intrapleurally using a serial administration protocol twice daily over three days resulting in 12 instillations [3]. The study results have become a mainstay of CPEE management. It is, however, time-consuming, requires trained staff to administer medications four times daily, and to open the chest tube for drainage additional four times daily. The non-adherence to the protocol was 29% [3].

Since the publication of MIST 2 study, there is an interest in the concurrent administration of tPA and DNase and tailoring of the protocol to improve adherence and reducing complications [5-7]. We reviewed these studies and added our experience of concurrent administration of tPA and DNase for CPEE. Due to a large contingent of thrombocytopenic patients, we have tailored our protocol to 10mg of DNase and 4mg of tPA, as standard practice. Our hypothesis behind the dose alteration is in essence that tPA is associated with a majority of the bleeding complication of the regimen since DNase can't really be effective unless it has access to the DNA.

## Materials and methods

We reviewed the records of patients who were given intrapleural tPA and DNase for complicated parapneumonic pleural effusion and empyema between November 2014 and February 2016. Electronic medical records were reviewed for patient demographics, pleural fluid characteristics, laboratory data, chest tube size, the timing of chest tube insertion and medication administration, the total duration of treatment, and patient outcomes.

CPEE was defined based on the American College of Chest Physicians (ACCP) consensus guidelines [8] and in line with previous studies [5-7] as a pleural fluid with any of the following: (1) exudative and loculated in a patient with community or hospital-acquired pneumonia, (2) gram stain or culture positive, and (3) macroscopically purulent. Pleural fluid pH is not routinely measured at our institution and is not used as criteria for chest tube insertion. Exclusion criteria were pleural space infections as a complication of a procedure/operation, age below 18 years, or patients who had a chest tube inserted prior to hospitalization at our institute.

All patients underwent chest tube drainage. The decision to insert a chest tube, the dosage, and when to initiate intrapleural tPA/DNase therapy was determined by the attending physician. Loculation was determined bedside by sonography and/or computed tomography (CT) scan. Medication doses were tPA at 4mg and DNase at 10 mg. The drugs were delivered at the same time followed by 10 ml of saline flush. The chest tube was clamped and the medications were allowed to dwell for two hours before the chest tube was opened for drainage with 20 cm of suction. Selection of the timing of treatment, number and doses of treatments, and removal of chest tube were per the attending physician's judgment.

Treatment was considered a success when patients were discharged alive without surgical intervention and with evidence of clinical and radiologic improvement of pleural space infection prior to hospital discharge. Clinical improvement was defined as improvement in vital signs, normalization of inflammatory markers, and improvement in the patient’s symptoms. Radiologic improvement was defined as a decrease in pleural opacification on follow-up imaging studies after intra-pleural treatment. Treatment failures were defined as patients requiring surgical intervention or death before hospital discharge. We also describe the number of treatment and doses given for each patient, the length of hospital stay, and 30-day mortality.

Patient characteristics were summarized using counts and percentages of categorical variables. Statistical analysis was performed on a per-patient basis. The length of hospital stay, treatment, and chest tube duration are presented as averages or as medians. All analyses were performed using SPSS.

We searched MEDLINE database (National Library of Medicine, Bethesda, Maryland) for MeSH terms such as empyema, intrapleural therapy, TPA and DNase. We were able to identify four studies involving concurrent administration of tPA and Dnase [6-9]. Out of these, one was a non-human subject study and was excluded [12]. Full texts of all three studies were available and reviewed in detail.

## Results

A total of 17 consecutive patients received intrapleural tPA and DNase during the study period. Two patients were excluded because the chest tube was placed prior to the current hospitalization. Fifteen patients met the inclusion criteria. Baseline characteristics of the patients are summarized in Table 1.

**Table 1:** Demographics and baseline characteristics of patient with CPEE treated with co-administered tPA/DNase CPEE: complicated parapneumonic effusions empyema; tPA: tissue plasminogen activator; DNase: deoxyribonuclease

Age in years (n=15)	60.1
Sex (n [%])	
Female	1 (6.7)
Male	14 (93)
Comorbidities [n (%)]*	
0	5 (33.3)
1	5 (33.3)
2	4 (26.7)
3	1 (6.7)
Admit location (n [%])	
Floor	7 (46.7)
ICU	7 (46.7)
ED	1 (6.7)
Loculated pleural effusion [n (%)]	13 (86.7)
Gram stain positive [n (%)]	6 (37.5)
Culture positive [n (%)]	6 (37.5)
Time to intrapleural tPA/DNAse after CT insertion [n(%)]	
Within 2 days	9 (60)
After 2 days	6 (40)
Chest tube size [n (%)] (Fr)	
Less or equal 14	9 (60)
More than 14	6 (40)

The patients were older and majority of them were men. Most of the patients had atleast one comorbidity. Comorbidities that were listed include diabetes mellitus, chronic kidney disease, chronic obstructive pulmonary disease, asthma, pulmonary tuberculosis, congestive heart failure, history of malignancy (active or treated), stroke, hepatitis, and HIV.

Pleural fluid was loculated in the majority of patients (n=13, 87%), gram stain and cultures were positive in six patients. Streptococcus species was most commonly isolated, either alone or with another organism. One patient had *Staphylococcus aureus* in addition to streptococcus species. The majority of patients was managed with an image-guided 14-Fr or smaller pigtail chest tube and received intra-pleural tPA and DNase within 48 hours of chest tube placement. Median length of stay (LOS) was 18 days. Median length of chest tube dwell time was 8.5 days.

Outcomes are shown in Table 2.

**Table 2:** Individual patient and treatment data tPA: tissue plasminogen activator; DNase: deoxyribonuclease

	Age	Sex	Admit to chest tube Insertion (d)	Admit to first thrombolytic dose	Chest tube (d)	Hospital length of stay (d)	Chest tube size (Fr)	No. DNase doses	Total amount of TPA/Dnase	Outcome on discharge day
1	53	M	16	19	8	23	16	3	4/30	Alive
2	63	M	5	5	6	15	10	2	8/20	Alive
3	82	M	2	3	4	15	14	1	4/10	Alive
4	67	M	1	4	9	10	14	3	12/15	Alive
5	69	M	101	103	6	107	12	2	12/15	Dead
6	81	M	4	16	19	39	14	1	10/5	Dead
7	53	F	30	30	30	45	16	4	12/35	Alive
8	56	M	5	6	9	14	10	2	30/10	Alive
9	26	M	32	34	10	43	16	7	70/35	Alive
10	59	M	3	11	15	20	16	5	24/50	Alive
11	58	M	7	15	10	18	16	1	5/10	Alive
12	78	M	25	25	1	27	16	1	5/10	Alive
13	49	M	0	1	5	11	16	2	8/20	Alive
14	65	M	0	3	8	9	16	1	4/10	Surgery
15	43	M	1	3	3	9	14	2	8/20	Alive

Twelve patients (80%) were discharged alive, one patient (7%) needed surgery and two (14%) patients died. The patient that required surgery underwent video-assisted thoracoscopic surgery (VATS) and decortication. This patient had VATS decortication after the first intrapleural treatment. Two patients died during their hospitalization due to unrelated causes. One had metastatic sarcoma and succumbed to enterococcus bacteremia and the other developed candidemia during hospital stay from central venous line, which proved fatal.

There were no complications related to the use tPA/DNase. No drop in hemoglobin or possibility of hemorrhage was observed. Three patients complained of chest pain, which was controlled with pain medications. Mean tPA dose was 14.1±17 mg/patient (median dose 4 mg/pt) and mean DNase dose was 19.7 ± 12.2 mg/patient (median dose 10 mg/pt). (Table 2).

Review of the currently available four case series with concurrent administration of tPA and DNase demonstrates successful clinical and/or radiological resolution in 89% (80%-92.7%.) of the cases. Pleural fluid characteristics were significant for loculation in most patients (77%-100%). The pleural fluid was gram stain or culture positive in 26%-62% of the cases. The median dose of tPA varied from 4 mg to 10 mg and DNase 5 mg to 10 mg; however, ours is the only study with a different dose of lytic medications used, and no hemorrhagic complications. The median number of concurrently administered doses ranged from two to six although only one study reported six doses, while two studies reported median number of doses two and one reported three, that is much lower than six doses in MIST 2 trial. Median chest tube dwell time was from 5 to 9 days and median length of hospital stay was from 11 to 18 days. Clinical and/or radiological improvement was seen in 89% across all four studies (80% to 93%). Surgical intervention was required in 8% (7%-10%), and mortality due to pleural infection was reported in 1.6%. Hemorrhagic conversion occurrance was 2.6% (Table 3) [5-7].

**Table 3:** Review of studies with concurrent administration of tPA and DNase tPA: tissue plasminogen activator; DNase: deoxyribonuclease

	Current study	Majid et al.	Mehta et al.	Bishwakarma et al.	All
Total patients	15	73	55	39	191
Loculated effusion	13 (86%)	56(77%)	n/a	39 (100%)	-
Gram stain/ cx positive	6 (40%)	19(26%)	34 (62%)	23 (59%)	85 (45%)
tPA dose	4 mg	10 mg	10 mg	10 mg	-
DNase dose	10 mg	5 mg	5 mg	5 mg	-
Median number of doses	2	2	3	6	-
Median time from chest tube to intra-pleural treatment	2	1	1	2* (within 2 days)	-
Median chest tube dwell time	8.5	5	9	8.6	-
Median hospital stay	18	11	13	14.5	-
Clinical/radiological Improvement	12 (80%)	66(90%)	51(93%)	33 (85%)	170(89%)
Required surgery	1 (7%)	7 (10%)	4 (7%)	3 (8%)	15 (8%)
Mortality due to pleural infection	0	2 (3%)	0	1 (2%)	3 (1.6%)
Non-empyema related death/LOC change	2	n/a	3	2	-
Increased pain	3	11	8	n/a	
Hemorrhagic conversion	0	4	0	1	5 (2.6%)

## Discussion

Intrapleural therapy has been found to improve drainage of CPEE [3]. MIST 2 trial has established intrapleural therapy as the mainstay of CPEE treatment hence avoiding surgery and decreasing the length of hospitalization [3, 8, 10-12]; however, little is known about the correct dosage needed for tPA and DNase [6]. Dose and duration of intrapleural therapy based on MIST 2 involve multiple dosing and can be time-consuming for health care providers [3, 6]. Previous studies showed that complexity of treatment is a factor associated with poor adherence to a regimen [9]. For this reason, trying to find the minimum effective dose and simplifying the regimen is essential for minimizing side effects and maximizing adherence. The review of currently available literature and our center’s experience shows concurrent administration of tPA and DNase to be safe and effective even at lower cumulative dose. Majid et al. reported 80.8% of their study cohort was effectively treated with fewer than six doses of tPA/DNase therapy [6]. Mehta et al. showed once-daily serial administration of tPA and DNase in retrospective series was safe and effective [7].

Patients in our case series received a lower cumulative dose and lower number of doses with similar efficacy and without any complications, including pain and hemorrhagic conversion. It is possible that a higher dose of DNase might have increased the treatment efficacy without adding to the complications. Patients in our case series were older than previous studies and the median hospital length of stay of 18 days was longer than the hospital stays of patients in MIST 2 of 11.8 days [3]. Also, it was longer than the hospital stay reported by Mehta et al. [7] with 13 days and by Bishwakarma et al. with 14.5 days [5]. The chest tube dwell time had a median of 8.5 days. The fact that our institute is a tertiary care hospital in a state which has lower health literacy and is resource poor as compared to the states where the other studies were based leads to patients taking longer time to seek medical assistance and are sicker on arrival. The patients also lack resources themselves; four patients in the study could not be discharged from the hospital in a timely manner due to lack of transport. All these factors are likely to impact the length of stay.

Inclusion criteria included all patients regardless of expected prognosis or comorbidity. By this more liberal inclusion criteria, we present a more realistic estimate of safety, efficiency, and mortality.

Overall, the median number of doses and the median cumulative dose in three studies was lower than the MIST 2 trial and equal in one study [3-7]. Although it is difficult to make direct comparisons, the results in this paper appear to be similar to previous studies using MIST 2 protocols and dosing. For this reason, we believe more research may need to be conducted to establish the optimal dose of the combination intrapleural therapy. There is an American Association of Bronchology and Interventional Pulmonology sponsored randomized controlled trial (RCT) underway by Majid et al. comparing concurrent to sequential instillations. We plan to propose an RCT comparing our protocol of higher dose of DNase and a lower tPA dose to the MIST 2 dose regimen.

## Conclusions

It is clear that a combination of tPA/DNase is needed for the management of CPEE. It is, however, less clear as to which dose to use and how frequently the treatment needs to be provided. Our review of the literature and case series demonstrates that a simpler concurrent administration of a lower dose of intrapleural tPA and DNase is safe and effective for CPEE.
